# How Helpful May Be a CRISPR/Cas-Based System for Food Traceability?

**DOI:** 10.3390/foods13213397

**Published:** 2024-10-25

**Authors:** Silvia Farinati, Aurélien Devillars, Giovanni Gabelli, Alessandro Vannozzi, Francesco Scariolo, Fabio Palumbo, Gianni Barcaccia

**Affiliations:** Department of Agronomy, Food, Natural Resources, Animals and Environment (DAFNAE), Campus of Agripolis, University of Padova, Viale dell’Università 16, 35020 Legnaro, Italy; silvia.farinati@unipd.it (S.F.); aurelien.devillars@phd.unipd.it (A.D.); giovanni.gabelli@unipd.it (G.G.); alessandro.vannozzi@unipd.it (A.V.); francesco.scariolo@unipd.it (F.S.); fabio.palumbo@unipd.it (F.P.)

**Keywords:** genome editing, new genetic techniques, new breeding techniques, molecular traceability, CRISPR/Cas system

## Abstract

Genome editing (GE) technologies have the potential to completely transform breeding and biotechnology applied to crop species, contributing to the advancement of modern agriculture and influencing the market structure. To date, the GE-toolboxes include several distinct platforms able to induce site-specific and predetermined genomic modifications, introducing changes within the existing genetic blueprint of an organism. For these reasons, the GE-derived approaches are considered like new plant breeding methods, known also as New Breeding Techniques (NBTs). Particularly, the GE-based on CRISPR/Cas technology represents a considerable improvement forward biotech-related techniques, being highly sensitive, precise/accurate, and straightforward for targeted gene editing in a reliable and reproducible way, with numerous applications in food-related plants. Furthermore, numerous examples of CRISPR/Cas system exploitation for non-editing purposes, ranging from cell imaging to gene expression regulation and DNA assembly, are also increasing, together with recent engagements in target and multiple chemical detection. This manuscript aims, after providing a general overview, to focus attention on the main advances of CRISPR/Cas-based systems into new frontiers of non-editing, presenting and discussing the associated implications and their relative impacts on molecular traceability, an aspect closely related to food safety, which increasingly arouses general interest within public opinion and the scientific community.

## 1. Introduction

Our daily lives are largely influenced by products derived from biotech-based technologies, whether for industrial and medical purposes or for food and feed use, although most people are not even aware of it. In fact, the food and agricultural industries are quickly integrating a variety of novel genetic technologies. In addition to traditional plant breeding methods, an increasing range of approaches, including genetic engineering and contemporary biotechniques, are becoming available for selecting and introducing desirable features in crop plants and consequently in the food products derived from them. The potential application of these different strategies in agriculture, but not only, is a current new trend, and in particular, the possibility of precisely modifying the genome has had an extraordinary social impact since its origin in 2012 [1]. This new approach combines a number of characteristics that significantly influence biotech-based approaches in general and in agricultural sectors, like *in primis* high precision and flexibility. Crop plants obtained by recent genome/gene editing (GE) technologies represent a notable shift away from genetically modified or transgenic plants [2,3]. In general, the global discussion is centered on regulations pertaining to the use of gene editing in the food-related sector. Although consensus-building and public acceptance of new policy measures are frequently challenging, it is important to provide quantitative data regarding public opinion of these developing technologies. It was therefore necessary to ‘quantify’ and make this perception more analytical through the formulation of empirical models, as demonstrated by two web-based surveys carried out between December 2016 and February 2017 in Japan. Based on their levels of domain-specific scientific expertise, participants (N = 3197) were divided into specialists in molecular biology, experts in other domains, and the general public. Group disparities in the risk, benefit, and value assessments of various technologies were found by statistical analysis [4]. However, one of the main goals in public and/or private contexts is to highlight possible conceptual and technical ambiguities, often due to the lack of simple definitional boundaries among different applicable approaches. During the last decade, the European Commission (EC)’s Group of Chief Scientific Advisors compared the traditional breeding methods, the well-established genetic modification (GM) methods, and the new genomic technologies (NGT), also known as new breeding techniques (NBT), proposing the following conclusions: (i) realistically, safety assessments can only be made on a case-by-case basis and depend on the characteristics of the end product; (ii) genetically and phenotypically similar products resulting from the use of different techniques are not expected to present significant risks [5]. In particular, regarding the application of NBTs based on GE approaches, clear guidelines rather than border lines should be necessary to highlight and define how the institutions intend to outline the concept of breeding strategies in view of these new emerging biotechnologies as alternatives to conventional GM crop development, despite the fact that GM production is still a major world power today. In 2017, the GM-based agricultural industry celebrated its 22nd year of commercialization, with 189.8 million hectares of GM crops farmed in 24 countries, including 19 developing and 5 industrialized nations. Compared to industrialized nations, which grow 47% of the world’s biotech crop area, developing nations grow 53%, and 40 more nations officially import biotech crops for use in food, feed, and processing [6]. In 2022, the global area planted with GM crops reached a new record of 202.2 million hectares, up 3.3% from the year before. Eleven distinct GM crops were grown in 27 different countries, with 98.9 million hectares planted with soybeans, followed by maize at 66.2 million hectares. Since the initial release of genetically modified crops in 1996, there has been a fluctuation in the number of nations growing GM crops [7]. For these reasons, concurrently with the advent of new editing technologies, a deeper delineation has become even more necessary to establish in a clearer and more defined way the boundary of what is or is not feasible and permitted from each specific governance. In fact, in addition to the administrative and legal aspects, increasingly frequent questions are becoming more common among the public; in particular, is there a real tangible advantage of these GE-based technologies compared to conventional breeding approaches or methods generally used for the GM crop production? Moreover, how can a technology based on genome editing be useful and exploited in a molecular traceability context? Is it possible to use these new technologies and derived ones in this applied research area with direct repercussions for the safe food industry in agri-food? Answering all these questions unambiguously results in being extremely complicated since the topic develops into an articulated scenario with a global interest.

In the attempt to answer and starting from the assumptions, we reviewed and outlined the main aspects related to the general use of GE-based approaches, considering both their best-known roles in target editing of the genome and their new frontiers with specific reference to no editing implications, including the impacts (and influences) on the molecular traceability in the agri-food sector. In particular, the main objective is to highlight and emphasize the technical features that enable the GE systems to be extremely adaptable and make them useful in a variety of contexts. In detail, the manuscript would provide an overview and focus attention on the main advances of CRISPR/Cas-based systems into new frontiers of non-editing, presenting and discussing the implications on molecular traceability, an aspect related to food safety, which increasingly arouses general interest within the public opinion and the scientific community.

## 2. Genome-Editing Toolbox as a New Frontier of Precision Breeding of Food Crops

The term genome editing reflects one of the most revolutionary and well-established strategies used in agricultural biotechnology and crop breeding in recent decades. Multiple aspects of GE have contributed to the advancement of modern agriculture and led to shifts in market structure because of the introduction of targeted changes within the existing genetic blueprint of an organism. Generally, the toolbox on which it is based is composed of various techniques that can be used to incorporate targeted, precise modifications into an organism’s genome [8]. Apart from the viability of the strategy itself, the ethical issue around GE in breeding is intertwined with a broader moral assessment of contemporary agriculture and its social and ecological legitimacy. GE, as a relatively new technology, is gaining importance as a tool for crop improvement because of its advantages over routinely used methods of genetic engineering available to produce GM organisms. GE approaches are technically precise and efficient, and the diversity of functions demonstrated allowed for successful gene therapy applications in yeast, animals, human and nonhuman cell lines, and embryos. All aspects at the base of gaining trust in public opinion [9].

The three foundational GE technologies that have been developed and improved in recent decades include transcription activator-like effector nucleases (TALENs), zinc-finger nuclease (ZFN) technology, and clustered regularly interspaced short palindromic repeats (CRISPR)-associated protein (Cas) technology [10]. These gene editing tools are based primarily on the enzymatic activity of a protein complex able to cleave DNA following direct or indirect recognition of a specific DNA sequence in the genome, cleave it, and on cell endogenous DNA repair machinery activity [8]. Currently, among these, the CRISPR system has become the dominant inducer method of GE, sweeping aside most other previously developed ZFN and TALEN tools. Indeed, since it is easily programmable and highly versatile to target the nuclease to the desired location in the genome, it results in the system that is by far the most widely employed for GE applications [11,12]. Furthermore, since the CRISPR/Cas-based genome editing system was developed from a prokaryotic adaptive immune approach against certain viruses, it was introduced to the scientific community as only a modification of a natural mechanism. In addition, CRISPR/Cas-based technologies represent a considerable improvement over prior techniques, as they are simple and versatile in terms of vector design and construction for subsequent plant transformation, and they are regarded as more reliable and straightforward for targeting gene editing for several purposes [13]. CRISPR/Cas has proven useful in a variety of sectors, including clinical diagnostics, food safety, and biological breeding. Because of their high specificity, sensitivity, and ease of use, class 2 systems, which include Cas9, Cas12a (Cpf1), Cas12b, Cas13a (C2c2), and Cas14a (Cas12f1), are currently the most studied and used single-protein effectors for numerous techniques [14].

From a structural point of view, the CRISPR/Cas-based system consists of a short noncoding guide RNA (gRNA) made up of two basic elements represented by a target complementary CRISPR RNA (crRNA) and an auxiliary transactivating crRNA (tracrRNA) [15]. The gRNA can guide the specific Cas endonuclease protein linked to it towards a specific genomic locus via base pairing between the crRNA sequence and the target sequence and cleave the DNA to create a double-strand break [16]. In bacteria, CRISPR loci are composed of a series of repeats separated by segments ~30 bp in length of exogenous DNA called spacers. The repeat-spacer array is transcribed as a long precursor and processed within repeat sequences to generate small crRNAs that specify the target sequences, also known as protospacers, following cleavage by the Cas9 protein. CRISPR spacers are then used to recognize and silence exogenous genetic elements at the DNA level. Essential for cleavage is a three-nucleotide sequence motif (NGG) immediately downstream of the 3′ end of the target region, known as the protospacer-adjacent motif (PAM) [15].

**Figure 1:**
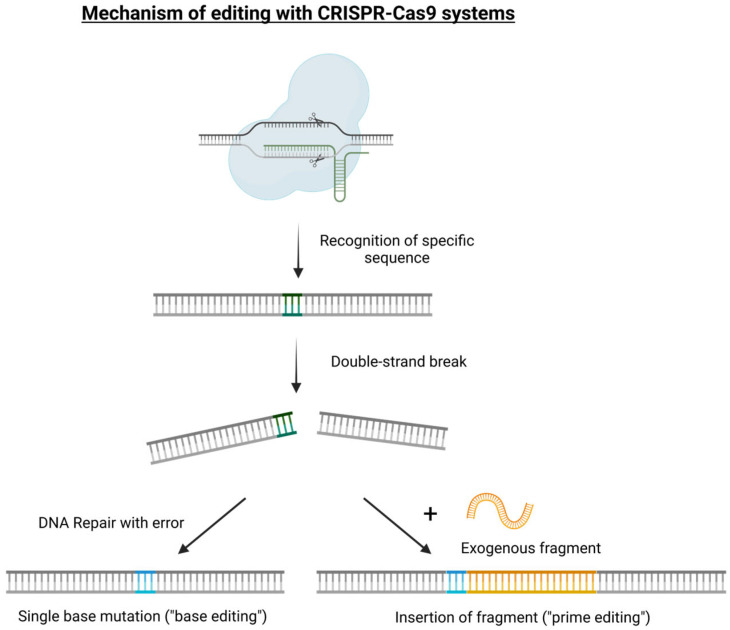
Simple graphical representation of the main genome editing mechanisms based on the CRISPR/Cas9 tool: single-base mutation (“base editing” for gene knockout or silencing applications) and exogenous fragment insertion (“prime editing” for gene replacement applications). The Cas9 recognizes the target sequence thanks to the specific gRNA it is carrying. The two RuvC nuclease domains of the Cas9 each cleave one strand of the sequence, leading to the formation of a double-strand break in the target sequence, which will be repaired by the NHEJ complexes, sometimes generating point-mutations, called in that case base edition. In the case where an exogenous fragment whose extremities match with the region of the double-strand break is present in the cell contemporaneously to the cleavage, it may be incorporated by the HR system at the place of the double-strand break, allowing for what is called prime editing.

Several Cas enzymes have been put into use because of their peculiar nuclease activities. Because they originate from various CRISPR systems, they may be beneficial for editing techniques due to their slightly varying abilities to recognize and change PAM sites [17,18]. In general, the merits of these systems include swift, broad acceptance for editing applications in a wide variety of plant species, as confirmed by an increasing number of studies in which an expanded use of Cas9 nuclease for editing beyond double strand breaks has been applied [19,20,21]. Errors occur during the subsequent repair step, making the DNA not identical to the original DNA. This allows for the insertion of a permanent minor change to the DNA, known as a mutation. Alternatively, additional variants of CRISPR/Cas-based technology have been developed, supporting the precise editing of a single nucleotide (“base editing”) or rewriting a given sequence of DNA (“prime editing”), as in the case in which a DNA template fragment can be added and introduced in association with the gRNA and Cas protein (Figure 1). With these strategies, a DNA template can also be used to introduce foreign genes and sequences into the genomes of plant species, leading to the formation of a transgenic crop but avoiding any positional effect [22]. In plant research, this technology is currently exploited for gene knock-out (loss-of-function) or gene replacement (gain-of-function) and it is referred to as precision-type plant breeding technology, actually representing a technology-assisted plant evolution strategy.

As previously anticipated, the scientific community, supported by specific legislation inherent, is rapidly increasing the utilization of NGTs to exploit their potential in designing new genotypes or specifically adjust plant traits. Since these new technologies are often adopted for breeding programs, the terminology NBTs overlaps in some cases [23,24,25,26]. As evidence of this, several approaches testified numerous successes in a significant number of food crops, with direct repercussions on germplasm resources [26]. However, not only: the CRISPR/Cas9 system has been shown to be efficiently applied in restoration [27], either to modify or upregulate key functional genes or to engineer synthetic gene drives for spreading positive or negative genetic elements throughout populations, with the potential development of novel genotypes to thrive under challenging conditions and gene drives to help control pest species [28].

Many reviews and investigations have addressed the fundamentals and different aspects of using CRISPR/Cas in functional genomics and crop improvement, particularly in regard to producing target mutants of the genes responsible for several traits related to agronomical characteristics and/or physio-biological responses. Applications have mostly concentrated on characteristics linked to disease resistance, environmental stress tolerance, food quality enhancement, and increased crop yields with few inputs [13,19,20,29,30,31]. All this progress has also been made possible by the development of high-throughput sequencing (HTS) platforms that promote and support this study of gene regulators of important traits or specific responses to the environment, as attested by many studies in which the enormous potential of editing approach-based technology has been reported. The HTS platforms played a key role also in the prediction of potential off-argets, namely the insertion of unanticipated, undesired, or even harmful changes to the DNA, representing a major concern in the applications of the CRISPR/Cas-based system. Numerous algorithms have been developed so far for identifying or locating CRISPR/Cas off-target sites, which provided the framework for the successful modification of CRISPR/Cas derivatives. The possibility of predicting any off-targets *in silico*, generally through open-source online software conveniently accessed via the internet, certainly represents a clear favorable point of the entire technology, despite these computational methods being usually insufficiently considering the complex intranuclear context (i.e., chromatin organization states); thus, off-target prediction by *in silico* tools needs further validations [32,33].

At a practical level, numerous successes may be registered, ranging from the examination of stress response control in roots at the cellular level to allele replacement for quantitative trait locus (QTL) validation, all traits related to roots crucial for crop improvement utilizing CRISPR/Cas-based genome editing [34]. Additionally, developing cereal crops with root systems that can absorb irregularly distributed water and nutrient resources in times of resource scarcity and climate instability is another main application example of a primary breeding goal for CRISPR/Cas genome editing. To date, studies employing CRISPR/Cas-mediated strategies to increase crop abiotic stress tolerance have been the main research focus for food crop improvement using GE technology [35,36,37,38,39,40,41,42,43,44]; the development of crop resistance to pathogens (e.g., bacteria, viruses, and fungi); the modulation of targeting susceptibility systems, as demonstrated for the tomato yellow leaf curl virus in *Nicotiana benthamiana* [45,46,47], bean yellow dwarf virus in *Nicotiana benthamiana* [48], and beet severe curly top virus in Arabidopsis and *Nicotiana benthamiana* [21,47,49,50,51,52,53,54,55,56,57,58]. Furthermore, CRISPR/Cas technology has produced a successful substitute for the production of plant material that is resistant to a variety of herbicides; for example, CRISPR/Cas has been used to target the ALS gene in crops, including rice, wheat, and other cereals, and a number of herbicide-tolerant polymers have been created [59,60,61,62]. Finally, taking into account all these aspects, it is possible to consider the CRISPR/Cas technology as a potential turning point for global agricultural challenges like the improvement of crops cultivated in difficult climatic areas to gain resiliency against emerging pests and environmental stresses. The development of crop varieties with higher yields, better adaptability to the changing climate, and greater tolerance to biotic and abiotic stresses will be necessary in the next decade to keep up with the pace of population growth. In fact, according to recent estimates, in order to feed everyone on the planet, food production will need to rise by 50% by 2030 and by 70% to 100% by 2050 [63,64].

## 3. CRISPR/Cas Technology as a Precision Tool for Food Crop Traceability

**Figure 2:**
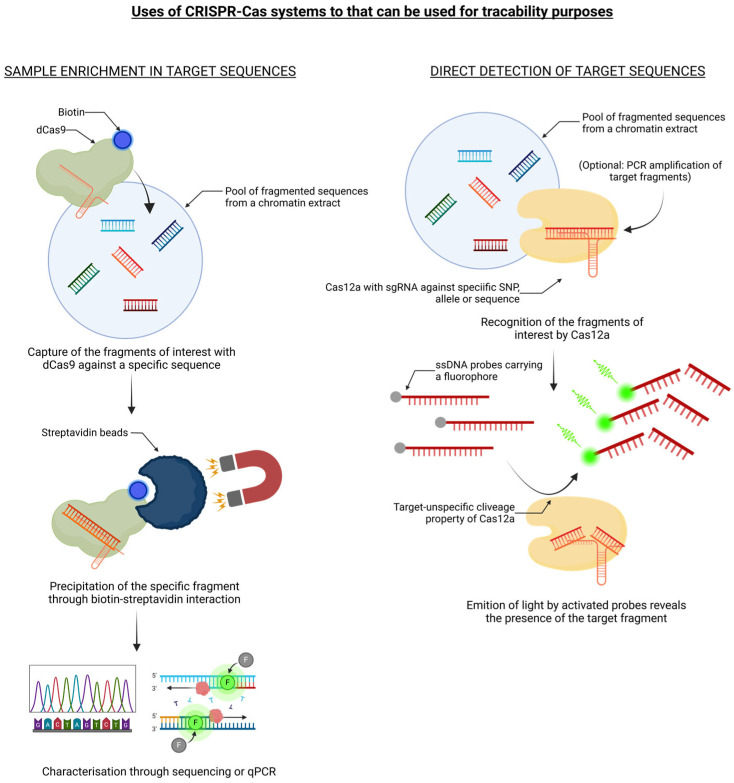
The CRISPR/Cas9 system can be used for molecular traceability purposes. Sample enrichment in target sequence: A pool of chromatin fragments is incubated with a catalytically inactive dCas9 fused with a biotin and carrying a specific gRNA. Afterwards, the dCas9 and the fragments that are bound to it are precipitated thanks to streptavidin-coated magnetic beads. Then, the nature of the precipitated chromatin fragments is assessed by DNA-sequencing or by qPCR. Direct detection of the target sequence: A pool of chromatin fragments is incubated with a catalytically active Cas12a fused with a biotin and carrying a specific gRNA along with single-stranded DNA probes associated with fluorophores. Recognizing its target sequence among the chromatin-pool activates the sequence-independent single-stranded DNA nuclease activity of the Cas12a, which will allow it to cleave the single-stranded DNA probes, which will induce the activation of the fluorophores, emitting a luminous signal that is interpreted as evidence of the presence of the target sequence in the pool. The details are reported in the text.

CRISPR/Cas technology, due to its enormous potential related to its precision and reliability, ease of use, and low cost, is now considered a valuable alternative to conventional plant breeding methods for intraspecific gene mutation (e.g., mutagenesis) or interspecific gene introgression (e.g., backcross strategy). As documented above, CRISPR/Cas has been used for an increasing number of applications, which has led to the development of a range of editing tools in crop plant science. However, since a genetic engineering approach may be affected by a highly contested and controversial societal issue, as was the case for conventional genetic transformation techniques (i.e., GMOs produced via transgenesis and intragenesis), the use of CRISPR/Cas as a new breeding technique poses new questions regarding the preferences of consumers and producers, food ethics, and political standpoints of governance leaders. Since its discovery in 2012 as a novel tool for genome editing, the CRISPR/Cas system has spawned a plethora of literature, but in-depth evaluations of its potentials and/or limitations in relation to food production and authentication by means of molecular or genetic traceability applications are just beginning to take shape. Indeed, these emerging genomic engineering approaches can offer a valid key for the effective prevention and control of food safety risks, in which the development of specific, sensitive, and rapid nucleic acid detection methods, such as foodborne pathogenic bacteria, genetically modified crops, and product adulteration, is imperative (Figure 2).

The sequencing of nucleic acids is the main approach for identifying one or more specific species of any kingdom since DNA acts as a natural biomarker for validation in both unprocessed food products and derivatives. The DNA barcoding technique, which refers to the identification of plant species using a short domain of the plastidial genome from a specific genic or intergenic target sequence, is currently available and still appears to be among the most useful food authentication applications based on DNA sequencing [65]. For similar reasons, recent isothermal amplification strategies and advances in DNA and omics-based technology (i.e., genomics, metabolomics, and proteomics) for the detection of specific nucleic acid regions have been developed, allowing for potential use in food testing, identification, and authentication [66,67,68]. Nevertheless, ascertaining food species and even varieties or genotypes by sequencing DNA barcodes or genotyping DNA markers is mainly performed through targeted polymerase chain reaction (PCR)-based and real-time quantitative PCR (qPCR)-based techniques coupled with methods to improve the specificity of PCR methods and increase the applicability and reliability/reproducibility of PCR for food authenticity applications [68,69,70,71].

Despite DNA barcoding being widely used as a routine molecular tool to date, at least when it comes to species-level identification strategies, there are still drawbacks to its use, including (i) the difficulty in creating universal primers for amplifying genomic regions that can specifically discriminate plant varieties or genotypes, and (ii) the low resolution of the technology when it comes to the identification of closely related species/botanical varieties/cultivars [72]. To overcome these limitations, the CRISPR/Cas system can now be used to detect food adulteration, mislabelling, the presence of allergens, and allergen sources due to recent technological breakthroughs, but also variety identification for food-destined crops [73]. The growing number of studies highlighting the potential of the CRISPR/Cas system in the investigation of food authenticity and its ability to improve PCR method detection performance is a testimony of the ongoing development of various technical solutions [74]. The combination of CRISPR/Cas with nucleic acid amplification techniques greatly improves the specificity and sensitivity of nucleic acid identification not only for food crops but also for detecting infections and nucleic acid biomarkers [75,76,77,78]. Several targeted approaches exploiting both collaboration and competition between CRISPR- and omics-based technologies have been developed, leading to the development of universal nucleic acid testing approaches based on the CRISPR/Cas system to identify DNA barcodes in a very sensitive and reliable way regarding food safety and authenticity. The resulting CRISPR/Cas-based DNA barcoding method showed great potential for food authenticity and broadened the application of the CRISPR/Cas system to these sectors [79,80]. Furthermore, most applications have demonstrated the potential of CRISPR/Cas systems to target nucleic acid portions due to the specific recognition of their sequence, which is also useful for non-editing applications. In the past decade, several new methods have emerged in the field of genome studies that involve the use of an interesting version of the Cas9 protein, referred to as dead-Cas9 (dCas9), which was developed to target DNA sequences through the use of a specific guide without cleaving them [81]. Indeed, this peculiar Cas9 version is deprived of its catalytic activity because of the two point mutations (D10A and H841A) that its amino acid chain harbors and that induce the loss of function of the RuvC1 and HNH nuclease domains [82].

In addition to the Cas9 protein, an alternative endonuclease, which is largely employed for these purposes, Cas12a, also known as Cpf1, is a typical V-A CRISPR system. Instead of producing blunt ends when it cleaves DNA, Cas12a can produce 5′ overhangs, which is more useful for some genome editing applications, such as inserting a DNA sequence at a particular point [83]. Furthermore, when activated by the highly specific binding of the DNA sequence indicated by the gRNA, Cas12a exhibits both sequence-independent single-stranded DNA degradation and sequence-specific double-stranded DNA cleavage [84,85].

### 3.1. CRISPR/Cas as an Enrichment Tool for Next-Generation Sequencing

Selective enrichment of nucleotide regions of interest using CRISPR/Cas technology for sequencing purposes has predominantly emerged in the traceability field due to its importance. The need to target specific genetic loci is crucial and, at the base of traceability purposes themselves, often conditioned/influenced by genome-wide approaches. In general, for the application of techniques based on genome-wide analyses, it is imperative to screen for signals from high-abundance undesirable species before sequencing [86]. The identification or enrichment of certain regions of interest has progressively increased as a crucial tool for traceable sequencing, prompting the creation of multiple procedures suitable for various experimental purposes. It is therefore presumable that in the coming years, additional tools that take advantage of the CRISPR/Cas system and its characteristics will be created in the scope of increasing the possibility of studying nucleic acid characteristics, such as the detection of structural variations at the haplotype level using long-read sequencing. These tools are mainly based on the high specificity of targeting through sgRNA and the existence of a plethora of available Cas proteins with the ability to perform a series of molecular actions on the nucleotide sequence of interest. For example, CRISPR-assisted targeted enrichment sequencing (CATE-seq) consists of the fragmentation and subsequent specific ligation of adaptors to sample DNA, after which the targets are bound by dCas9 and purified for allele-specific PCR or library preparation, resulting in a highly sensitive alternative approach able to reach over 3000-fold enrichment of the target sequences [87]. In addition, ultrasensitive CRISPR–Cas9-triggered nicking endonuclease-mediated strand displacement amplification (CRISDA) [88], which can achieve attomolar sensitivity, combines Cas9 cleavage activity with highly specific target site amplification and annealing of biotin and Cy5-labelled PNA (a peptide nucleic acid) probes to the amplicon. In contrast, CRISPR-mediated isolation of specific megabase-sized regions of the genome (CISMR) enables the isolation of regions of interest through Cas9-driven cleavage at flanking sites by combining a pulse-gel electrophoresis step for sequence isolation and an amplification step, followed by long-read sequencing [89].

### 3.2. CRISPR/Cas as a Detection System

As previously described, PCR-based methods, including qualitative PCR [90], quantitative PCR (qPCR) [91], droplet digital PCR (ddPCR) [92], and LAMP [13], have long been the primary and undisputed DNA detection strategies and have been utilized for traceability in all research sectors, despite being costly, labor-intensive, and time-consuming. Therefore, the improvement of rapid, simple, and sensitive new detection methods for nucleic acids without special technical expertise and ancillary equipment is increasingly necessary.

The introduction of the CRISPR/Cas system as a novel gene editing tool has allowed biotechnology to enter a new era. In addition to the widely documented purposes, ranging from cell imaging to expression regulation and DNA assembly [93,94,95,96,97], a plethora of additional uses, defined as CRISPR/Cas-based nucleic acid detection system assays, have also been described, opening new possibilities that did not formerly exist or implementing performances of what existed but with a faster, cheaper, and easier strategy. To cite a few examples, the developed CRISPR-based tools, which could depend on amplification-based or amplification-free-based methods, provided several applications in authentication and detection of food products such as milk frauds and halal food, as well as the identification of plant ingredients or genetically modified species (see below).

In general, they mainly exploit the capacities of the Cas12a protein [76,85] to perform nonspecific ssDNA cleavage upon recognition of a target that matches its crRNA [16,83,94] and to produce a 5′ sticky end on the target itself [85,98,99]. Recently, recombinase polymerase amplification (RPA) and the nonspecific ssDNA cleavage property of Cas12a have been combined to create a novel detection technology platform known as RPA-Cas12a-FS: the combination of RPA with guide crRNA targeting and detection of FAMBHQ1-labelled reporter ssDNA allowed the development of an on-site detection method, and derived, for the molecular identification of various biological contaminants, genetically modified crops, and food adulteration [80,100,101]. For example, the substitution of goat’s or sheep’s milk with cheaper alternatives like cow’s milk was investigated, mediating a sensitive surface-enhanced Raman scattering (SERS) method, powered by Cas12a, for a rapid on-site milk fraud analysis [102].

Furthermore, in relation to Cas12 protein use, a new method combining CRISPR–Cas-based PCR DNA barcoding (CAPCOD) was developed, in which the specificity of the CRISPR/Cas12 system and the high amplification efficiency of the PCR method for identifying DNA barcodes to improve the accuracy of sample authenticity are combined [103]. The nonspecific endonuclease activity allows the random cleavage of adjacent single-strand nucleic acids by trans-cleavage upon recognition of the target sequence [93,104]; based on this understanding of its activity, CRISPR/Cas12 has been applied to nucleic acid testing because it can precisely identify amplicons via isothermal amplification or PCR [105,106,107]. This CRISPR-based DNA barcoding strategy resulted in a powerful tool for rapid authentication of halal food, offering high specificity and sensitivity in determining if the meat came from halal animals, such as cattle, sheep, or chickens. Indeed, exploiting the specificity of the Cas12 system and the high amplification efficiency of PCR, a specific RPA-Cas12a assay was employed for detecting pig-derived ingredients, thus indicating the potential for halal meat authentication. Additionally, the determination of plant ingredients takes on the same importance for identifying potential allergens in food items [108]. Currently, tests that use the CRISPR/Cas systems for plant component authentication are on differentiating between bulk and fine cocoa, based on a Cas9-based assay for identifying a single nucleotide polymorphism (SNP) [101]. Since CRISPR/Cas can be considered an effective method for molecular detection based on trans-cleavage activity, this ultrasensitive detection method can usually be exploited for the detection of GMOs, gene-edited products, and SNPs [80,100,109]. Regarding GMO detection or edited products, with and without exogenous sequences, the strategies may differ based on the employed approach. In contrast, the methods for detecting mutations of a single or a few bases can differ from those used to detect SNPs. A combination of LAMP and CRISPR/Cas12a was used for visual detection of GM soybean powders by UV light [110]. A portable biosensor for visual dual detection of the *CaMV35S* promoter and *lectin* gene in soybean powders, named Cas12a-PB, has also been developed [110]. Furthermore, in 2022, Cao and colleagues [111] established the MPT-Cas12a/13a technology, which combines multiplex PCR and transcription for simultaneous but distinct detection of *CaMV35S* and T-*nos* elements. Many different additional approaches have been established with unique analyses (particularly fluorescence detection and gold nanoparticle-based colorimetry assays) combined with CRISPR/Cas systems, and they have been extensively reviewed by Wang et al. [18].

For edited sequences in which universal components cannot be identified in the same manner as for GMOs, it is necessary to pick a specific motif with an appropriate PAM site for subsequent detection. An interesting method, called PCR/ribonucleoprotein (RNP), to detect mutations in gene-edited diploid and polyploid plants is to assemble CRISPR/Cas9 and CRISPR/Cpf1. This method is able to differentiate between homozygous and biallelic mutations as well as mutagenesis induced by TALEN protein and mutant screening that is unaffected by background noise SNPs, resulting in particularly advantageous results for screening polyploid plants [112]. In rice, a Cas12aFVD platform for biosensing and for the visible detection of gene-edited mutants was also developed [113]. For gene-edited products with known editing sites and sequences, methodologies like ACT-PCR, ddPCR, AS-PCR, CRISPR/Cas, etc., can be used for preliminary screening, whereas in the presence of known editing sites and sequences, they can be detected according to the current GMO detection strategy. In contrast, for gene-edited products with unknown editing sites and sequences, one or more methods can be used for preliminary screening. In general, Sanger sequencing, NGS, T7EI, and RFLP are currently the most extensively used methods; the use of other molecular-based techniques is less prevalent because the choice of detection techniques is strongly correlated with the kind of mutation, plant ploidy, and efficiency of gene editing [18,114].

The application of the CRISPR/Cas system for SNP detection has been employed with the purpose of providing different strategies that are useful for the discrimination of single nucleotide mismatches (SNMs). For example, when combined with asymmetric PCR, Cas12b successfully distinguished the SNP locus without the PAM sequence. This means that it can cleave ssDNA without a PAM sequence [94]. Several other reports have confirmed the success of different combination strategies for distinguishing SNPs and achieving single-base resolution detection in all kingdoms [78,115,116].

CRISPR/Cas detection systems can also benefit from the application of microfluidic technology. In 2021, Chen et al. [109] included a nucleotide mismatch to increase the universality of SNP detection. To automate the procedure, CRISPR/Cas12a reagents were preloaded onto the biochip to differentiate between the homozygous wild type, homozygous mutant type, and heterozygous mutant type strains.

These innovative methods offer valid alternative tools for DNA-level detection, assist as a front-line nucleic acid quick detection system for food safety, and may be applied at the end of the food inspection and genetic traceability or authentication industry chain. Regarding food safety issues, since nowadays it is common the addition of several kinds of chemical compounds to food, like chilly powders to deepen the red color, palm oil [117], formaldehyde to milk [118], synthetic flavors to fragrant rice, and so on [119], the setup of new methods, including CRISPR/Cas-based systems, for non-nucleic acid target detection is becoming increasingly important. These strategies result in very powerful detection of (in)organic compounds spanning from ions, antibiotics, pesticides, and veterinary drugs to heavy metal ions and secondary metabolites of pathogenic microorganisms (e.g., bacterial toxins, exotoxins, and mycotoxins). The basic theory on which such a principle is based is to translate non-nucleic acid into nucleic acid signals through exploiting signal transduction elements such as aptamer and responsive DNAzyme, often defined as (bio)recognition agents or in general as ‘antibodies of chemicals’ [120,121].

Despite all these CRISPR/Cas-based detections having reached extremely high levels of sensitivity and specificity in several topics, as revised in 2022 by Li and colleagues [122], the high general cost related to the use of sophisticated lab instruments and the common need for sample pre-treatments may constitute a potential limitation versus the feasibility to apply or implement them on a large scale in contexts outside (i.e., an open field) of a specialized laboratory.

## 4. Future Opportunities and Trends

This communication has the purpose of highlighting the wide application potential of the CRISPR/Cas technology as a support for molecular traceability purposes in several research fields related to crop production (i.e., plant varieties) and food commercialization (i.e., food products and their derivatives). However, despite the main purpose of the manuscript being to reveal this aspect of technology, it is not possible to ignore its key origin role in gene editing, the development of new varieties with higher yields, better adaptability to the changing climate, as well as being more tolerant to biotic and abiotic stresses, also in relation to the increase of global population growth. Through the CRISPR/Cas-based method, numerous genes have been edited, plant traits have been improved, and the resulting commercial items have been introduced into the market with direct social implications or opinions. The off-target identification and the evaluation of their potential effects remain again a major challenge, directing research forward novel strategies to improve CRISPR/Cas9 specificity and to reduce undesired mutagenesis for future applications. Since some long-term impacts result crucial for the biodiversity context, influencing germplasm resources and spreading positive or negative genetic elements throughout populations, with the potential development of novel genotypes, understanding the genetic basis of plant functional traits in detail is essential, requesting genomic studies and a well-annotated reference genome. CRISPR/Cas detection systems display numerous outstanding features, including low cost, easy operation, quick application, and high accuracy and reliability. However, most of the currently available detection methods are not suitable for prompt on-site detection because they require labor-intensive specialized methodologies, dedicated instruments, and sophisticated sample processing and analysis. In addition to gene editing, CRISPR/Cas systems have recently been employed for target detection and can also be used to efficiently identify proteins, metal ions, nucleic acids, and other chemicals via a variety of technologies. The high sensitivity and specificity of these methods are two of the highest strengths of the CRISPR/Cas system, and laborious experimental procedures and time-consuming analyses are not needed. To accomplish flexible detection of nucleic acid and non-nucleic acid targets in the domains of clinical diagnostics, environmental testing, biological breeding, food safety, and others, the CRISPR/Cas system can be coupled with a range of amplification techniques, readout techniques, and devices. Although CRISPR testing has been applied extensively, CRISPR nucleic acid testing remains in its infancy and has much room for development. CRISPR/Cas systems have many potential applications in the future due to research into their use with nanomaterials, 3D printing, the internet, big data, automation, and artificial intelligence. The industrial revolution and the biotechnological revolution are currently accelerating. Therefore, rapid, efficient, and accurate detection methods will become a major challenge for standard detection as more diverse traits and products continue to emerge, molecular characterization information and related databases are extremely limited and imperfect, and the optimization of mutation detection technology remains an ongoing attempt. This should be accomplished to guarantee researchers’ intellectual property rights, expedite the development of testing standards and procedures for biotechnology goods, and offer robust technical assistance for the oversight and monitoring of national security. As a conclusion, there is still much research work and dissemination to be performed to further improve and promote the NGT-based and derived systems in a context of food safety and traceability, but a wider social acceptance by the public opinion and a greater political reception of innovative technologies and policies by the governments are very promising starting points.

## Data Availability

No new data were created or analyzed in this study. Data sharing is not applicable to this article.
